# NDRG2 programs tumor-associated macrophages for tumor support

**DOI:** 10.1038/s41419-018-0268-8

**Published:** 2018-02-20

**Authors:** Silvia von Karstedt

**Affiliations:** 1 Department of Translational Genomics, University Hospital of Cologne, Cologne, Germany; 2 Cologne Excellence Cluster on Cellular Stress Response in Aging-Associated Diseases (CECAD), University of Cologne, Cologne, Germany

Cancer is a systemic disease and is, as such, much more than the sum of its malignant cells. For many solid tumors, this means that cancerous cells have found means to send signals to the immune system leading to immune evasion thereby allowing for uninhibited tumor growth. To date, a vast array of studies has made use of genetic knockout mice to understand roles of a given protein of interest in cancer. In many cases, however, it is vital to distinguish the function of this protein in cancer cells compared to its function in surrounding normal cells since this can prove to be the polar opposite. One such example is the role of the Myc-repressed gene N-myc downstream-regulated gene 2 (NDRG2) in cancer.

NDRG2 was first cloned from glioblastoma tissue, in which it was found to inhibit proliferation upon overexpression and to be downregulated during progression^1, 2^. Many studies followed in which NDRG2 was found to be downregulated in several types of cancers, an expression pattern that correlated with worse prognosis. In addition, overexpression of NDRG2 inhibited cellular traits thought to be cancer promoting^3^. Some mechanistic insight into how NDRG2 might function as a tumor suppressor came when NDRG2 was found to be a p53-inducible target gene with a direct p53 binding site which aided p53-induced apoptosis, whereas growth suppression induced by NDRG2 overexpression was independent of p53^4^. The crystal structure of NDRG2 revealed that, despite high structural homology to proteins of the α/β-hydrolase (ABH) superfamily, the conserved pocket required for enzymatic activity was absent. Instead, NDRG2 was shown to bind β-Catenin and thereby attenuate TCF/β-Catenin signaling^5^. Importantly, NDRG2-deficient mice spontaneously developed lymphomas and hepatocellular carcinomas as well as an overall increased activation of the AKT/phosphatidylinositol-3-kinase (PI3K) pathway, strongly suggesting this to be crucial for its tumor suppressor function in cancer cells in vivo^6^. Whereas these studies have clarified the function of NDRG2 in cancer cells and provided reasons as to why it is frequently downregulated in many cancers, its role in non-cancerous cells in the tumor microenvironment has so far remained elusive.

Now, a new study published in this issue of *Cell Death and Disease* has made the surprising discovery that NDRG2 in tumor-associated macrophages (TAMs) programs them for M2 polarization and thereby aids metastases outgrowth of NDRG2 wild-type (WT) cancer cells^7^. In a series of experimental metastasis experiments, the authors find that transplanting WT cancer cell lines into WT or NDRG2 knockout (KO) mice leads to reduced metastatic burden in the liver in the absence of NDRG2. Interestingly, reduced metastatic burden correlated with an increased percentage of M1-like TAMs and decreased M2-associated markers within metastases grown in an NDRG2-deficient environment. The authors then employ very elegant bone marrow (BM) depletion and reconstitution experiments to demonstrate that NDRG2 expression in BM is crucially involved in promoting metastases growth. Surprisingly, by doing these experiments they also uncovered another, yet again, tumor-suppressive function of NDRG2 in non-BM-derived cells of the liver microenvironment as underscored by the fact that reconstitution of NDRG2-deficient mice with WT BM not only reconstituted metastases growth to WT=>WT levels but even significantly promoted growth.

**Fig. 1:**
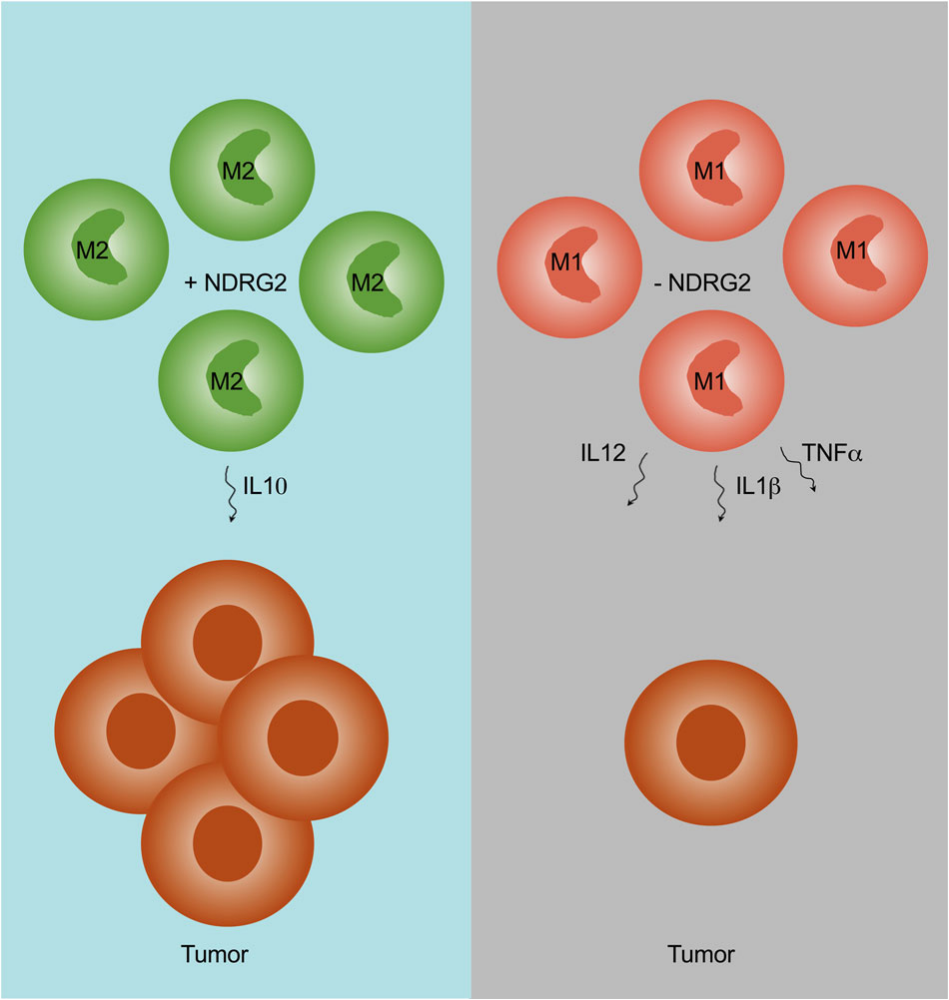
NDRG2 expression in TAMs facilitates their M2-like polarization (green). This results in the production of interleukin-10 (IL10), the promotion of a tumor-supportive microenvironment and tumor growth. The absence of NDRG2 in TAMs (red) in turn favors M1 polarization, production of IL12, IL1β and tumor necrosis factor-α (TNFα) and the suppression of tumor growth

Narrowing the tumor-promoting effect coming from BM-derived cells down to TAMs, the authors then performed tumor growth studies co-injecting tumor cells with NDRG2-KO or -WT TAMs. They found that, although green fluorescent protein (GFP)-labeled TAMs could not be detected anymore at the end of the study, the presence of WT TAMs but not NDRG2-KO TAMs at tumor initiation was sufficient to increase tumor size. These data together with previous findings demonstrate that while NDRG2 expression in TAMs favors M2 polarization and thereby facilitates tumor growth, its expression in normal liver cells as well as cancer cells serves tumor suppression. Mechanistically, they identify an increased and prolonged activation of the nuclear factor (NF)-κB pathway in NDRG2-deficient bone marrow-derived macrophages (BMDMs) to be required for M1 cytokine expression and polarization. Lastly, they demonstrate that NDRG2-deficient but not WT TAMs co-cultured with cancer cell lines without direct cellular contact (in transwell inserts) promote cancer cell migration, colony formation and invasion (summarized in Fig. 1).

Through a similar mechanism as identified for NDRG2 in TAMs by Li et al.^7^, fibrosarcoma and melanoma cell line migration and invasion was previously shown to be suppressed by NDRG2-mediated inhibition of the NF-κB pathway^8^. Importantly, NDRG2 was shown to suppress not only canonical but also non-canonical NF-κB activation by enabling NF-κB-inducing kinase (NIK) de-phosphorylation through recruitment of its phosphatase PP2A into a complex with phosphatase and tensin homolog (PTEN)^9^. At the same time, this recruitment was shown to allow for PTEN de-phosphorylation and activation and thereby a resulting inhibition of the AKT/PI3K pathway in the presence of NDRG2^6^. Confirming these results, a hyperreactive AKT pathway could also be observed in NDRG2-KO BMDMs in the new study by Li et al.^7^ making it highly likely that the NF-κB pathway in TAMs is suppressed by NDRG2 through a PTEN/PP2A/NIK axis. Although loss of NDRG2 expression as a clinical marker has mostly been associated with poor outcome in patients suggesting a tumor-suppressor role, a recent study has observed the opposite correlation for patients with basal-like breast cancer^10^. Since basal-like breast cancer cells can induce TAM M2 polarization^11^, it is tempting to speculate whether basal-like breast cancer is particularly dependent on NDRG2 to induce M2 polarization of TAMs to evade anti-tumor immunity.

Taken together, this study has for the first time shown a crucial and unexpected role for NDRG2 in TAM M2 polarization and at the same time highlighted the importance to investigate cancer as a systemic disease by studying gene function in vivo in cancer and normal cells within the tumor microenvironment.
